# Determinants of Patient Use and Satisfaction With Synchronous Telemental Health Services During the COVID-19 Pandemic: Systematic Review

**DOI:** 10.2196/46148

**Published:** 2023-08-18

**Authors:** Ariana Neumann, Hans-Helmut König, Josephine Bokermann, André Hajek

**Affiliations:** 1 Department of Health Economics and Health Services Research University Medical Center Hamburg-Eppendorf Hamburg Germany

**Keywords:** telemedicine, digital health, teletherapy, mental health, use, satisfaction

## Abstract

**Background:**

Several recent studies examined patient use and satisfaction with synchronous telemental health services in response to the widespread implementation during the COVID-19 pandemic. However, a systematic review of recent literature on the determinants of these outcomes is missing.

**Objective:**

The aim of this systematic review was to give an extensive overview of the literature on and highlight the influential determinants of patient use and satisfaction with synchronous telemental health services during the COVID-19 pandemic.

**Methods:**

This review satisfied the PRISMA (Preferred Reporting Items for Systematic Reviews and Meta-Analyses) guidelines and was registered in PROSPERO. Peer-reviewed, quantitative studies that observed the determinants of patient use or satisfaction with synchronous telemental health services during the COVID-19 pandemic were included. PubMed, PsycInfo, and Web of Science database searches were conducted in August 2022 for English and German language studies published from 2020 onward. Key steps were performed by 2 reviewers. Determinants were synthesized into major categories informed by the dimensions of the widely used and established Unified Theory of Acceptance and Use of Technology.

**Results:**

Of the 20 included studies, 10 studies examined determinants of patient use, 7 examined determinants of patient satisfaction, and 3 observed both outcomes. The quality of the studies was mainly good or fair. There was substantial heterogeneity in the study designs, methods, and findings. Sociodemographic characteristics and health-related determinants were mostly considered. Some of the major dimensions of the Unified Theory of Acceptance and Use of Technology were neglected in recent studies. Although most findings were mixed or nonsignificant, some indications for potential relationships were found (eg, for sex, age, and symptom severity).

**Conclusions:**

The findings revealed potential target groups (eg, female and young patients with mild symptoms) for future postpandemic telemental health interventions. However, they also identified patient groups that were harder to reach (eg, older patients with severe symptoms); efforts may be beneficial to address such groups. Future quantitative and qualitative research is needed to secure and expand on recent findings, which could help improve services.

**Trial Registration:**

PROSPERO CRD42022351576; https://tinyurl.com/yr6zrva5

## Introduction

### Background

Over the past 3 decades, health care services were usually delivered in person. Telemedicine is a promising, alternative service delivery model. The World Health Organization [1] summarized the four core characteristics of telemedicine as follows: (1) its purpose is to provide clinical support; (2) it is intended to overcome geographic barriers, connecting users who are not in the same physical location; (3) it involves the use of various types of information and communication technology; and (4) its goal is to improve health outcomes. Telemedicine benefits have been evaluated in the past and include, for example, reduced costs and improved access to services and information [2,3]. Evidence also suggests that telemedicine, in general, is a clinically and cost-effective tool with high satisfaction in patients and health care professionals [4]. However, the implementation of telemedicine has often been hindered by multiple barriers regarding reimbursement and clinical, legal, sustainability, and social issues [5,6].

In the wake of the COVID-19 pandemic, rapid changes in the delivery of health care services had to be made to prevent further spread of the virus, to protect people at higher risk of severe illness from COVID-19 (eg, patients with cancer, cardiovascular disease, or chronic respiratory disease), and to relieve the strain on the health care system. Consequently, telemedicine has been used worldwide across multiple specialties [7-9]. For instance, a large cohort study by Weiner et al [10] reported an increase in telemedicine use from 0.3% of ambulatory contacts between March and June 2019 to 23.6% between March and June 2020 among privately insured working-age individuals in the United States. Most telemedicine services were delivered via synchronous video or telephone calls during those periods [8].

The outbreak of the COVID-19 pandemic was also linked to stressors such as restrictions in everyday life, lifestyle changes, social isolation, and uncertainty and worries regarding health, finances, and work, which caused psychological burden [11]. Consequently, multiple studies have observed an increase in public mental health problems [12,13]. Liu et al [12] included 71 papers in their meta-analysis and detected an increased prevalence of anxiety (32.60%, 95% CI 29.10%-36.30%), depression (27.60%, 95% CI 24.00%-31.60%), insomnia (30.30%, 95% CI 24.60%-36.60%), and posttraumatic stress disorder (16.70%, 95% CI 8.90%-29.20%) during the pandemic. Moreover, preexisting mental health conditions were found to aggravate owing to the pandemic [14]. Therefore, patients with mental health conditions represented an especially vulnerable group during that time.

Telemental health services played an essential role in managing the increased public mental health burden and preventing the worsening of psychological symptoms. Mental health services are well suited for the remote format, as they do not require physical examination and can be delivered in multiple ways (eg, via telephone and video calls or mobile apps) [15]. In fact, telemental health services were found to be part of the medical specialty with the highest use rate during the pandemic [9]. The National Institute of Mental Health defined telemental health services as the use of telecommunications or videoconferencing technology to provide mental health services [16]. This can include synchronous (eg, videoconference and telephone) and asynchronous (eg, mobile apps and email) services. Regarding the effectiveness of telemental health services, an umbrella review of 19 systematic reviews on telemental health services before the pandemic suggested that remote mental health services produced at least moderate reductions in symptom severity and could be as effective as in-person formats [17]. They also found that user acceptance and satisfaction of telemental health services were comparable with those of in-person interventions. Recent reviews have also reported the effectiveness of and high patient and provider satisfaction with telemental health services during the pandemic [18,19]. Therefore, telemental health services seem to be a valuable addition to the treatment of mental illnesses of which implementation should be supported in the postpandemic future [20,21].

A crucial factor in the successful implementation of telemental health services is patient acceptance. In previous research, no universal definition of technology or telemedicine acceptance was identified. However, past definitions can be sorted into four main categories, which refer to (1) the effectiveness or efficiency of the services, (2) the use or adoption of the services, (3) the intention or willingness to use the services, and (4) consumer or provider satisfaction with the services [22-26]. To set a more precise focus, this systematic review concentrates only on patient use and satisfaction. In the course of this systematic review, patient use includes different measures of use behavior, such as the adoption of a new service, frequency of use, or attendance. Multiple definitions of patient satisfaction were introduced in the past and include various perspectives. For example, the expectancy-disconfirmation model defines consumer satisfaction as a function of expectation and expectancy disconfirmation, which can influence attitude change and purchase intention [27]. Although this definition is widely used, there is a lack of consensus regarding the definition of satisfaction [28]. The systematic review by Giese and Joseph [28] summarized three essential components of consumer satisfaction: (1) a summary affective response, which varies in intensity; (2) satisfaction, which focuses on product choice, purchase, and consumption; and (3) time of determination, which varies by situation but is generally limited in duration.

Different theories have been introduced to explain why patients accept telemedicine services. The Unified Theory of Acceptance and Use of Technology (UTAUT) [23] was thereby one of the most frequently used theories to predict patient acceptance of telemedicine [29]. In this theory, the key determinants of behavioral intention and technology use behavior are performance expectancy, effort expectancy, social influence, and facilitating conditions. In the context of telemedicine, performance expectancy is the degree to which an individual believes that using telemedicine could be helpful. Effort expectancy refers to the perceived ease of using the service, which also includes the effect of factors such as computer anxiety and computer self-efficacy. Furthermore, social influence means the degree to which an individual believes that others think that they should use telemedicine. Facilitating conditions include perceived organizational and technical infrastructure to support the use of telemedicine. Additional influential constructs in this theory include gender, age, experience, and voluntariness of use. User satisfaction was also found to be associated with major UTAUT constructs and to potentially contribute to the service reuse intentions [30,31].

### Objective

In addition to theoretical models, only few systematic reviews have summarized the determinants of patient use or satisfaction with telemental health services from prepandemic studies [32,33]. Potential determinants that were observed in these reviews were sex, age, education, socioeconomic status, living arrangement, cognitive function, experience with telehealth technology, comfort with using the internet, satisfaction with the health care provider, experience with the clinic, and cultural background [32,33]. Nevertheless, these reviews also highlighted the need for further research on this topic. The rapid, extensive implementation of synchronous telemental health services during the COVID-19 pandemic sparked international interest in the topic. Several studies examined the determinants of patient use and satisfaction with telemental health services since the pandemic. However, a systematic review of recent literature is missing.

Conducting such a systematic review may be helpful in identifying target groups, as well as groups that need further attention and support in relation to telemental health services. This could be of major importance to successfully implement postpandemic telemental health interventions and benefit from the remote format in the future, where it can be a valuable tool to deal with challenges, such as population aging (ie, shortage of health care professionals and increased demand for long-term care), stigma attached to visiting mental health facilities and undersupply in rural areas [34,35]. Moreover, it could be useful to identify gaps in the literature and guide future research. Therefore, the objective of this systematic review was to give an extensive overview of the literature on and highlight the influential determinants of patient use and satisfaction with synchronous telemental health services during the COVID-19 pandemic. In other words, this systematic review examined the following research question: what are the determinants of patient use of and satisfaction with synchronous telemental health services in studies conducted during the COVID-19 pandemic?

## Methods

### Overview

The systematic review protocol is available in PROSPERO (registration number: CRD42022351576). This manuscript was written in accordance with the most recent version of the PRISMA (Preferred Reporting Items for Systematic Reviews and Meta-Analyses) guidelines [36].

### Eligibility Criteria

For this systematic review, peer-reviewed quantitative studies in German or English that observed determinants of patient use or satisfaction with synchronous telemental health services during the COVID-19 pandemic were included. Only peer-reviewed quantitative studies were considered to assure high quality of the included studies. As most of the telemedicine services were delivered via synchronous services during the pandemic [8] and to assure comparability among the studies, only synchronous telemental health services were included. Mental health patients of all age groups (ie, children, adolescents, and middle- and older-aged adults) were considered to obtain as much information as possible from recent studies. Therefore, studies were excluded if they referred to (1) asynchronous services or eHealth interventions, (2) exclusively individuals with physical illnesses (to assure comparability among the samples), (3) data that were collected before the COVID-19 pandemic, (4) qualitative data, (5) outcomes that were not related to the use or satisfaction with telemental health services, or (6) studies that did not examine determinants of use or satisfaction with the services.

### Search Strategy

We searched the PubMed, PsycInfo, and Web of Science databases for studies published from 2020 onward. The PubMed and Web of Science databases are well established and frequently used in medical and related research fields. Moreover, they have also been recommended for searching telemedicine-related studies [37]. In addition, the PsycInfo database was included to account for the mental health context. A predefined search query was used to filter the databases (see Table 1 for the PubMed search query). Moreover, reference lists of eligible studies were screened for additional relevant articles. A pretest including 100 titles and abstracts was conducted before the screening process started.

**Table 1:** Search strategy (PubMed).

Serial number	Search term	Limits (filter, limits, and refine)
1	telepsychiatry OR online therap* OR telepsychology OR teleconferenc* OR teleconsult* OR online consult* OR videoconferenc* OR video consult* OR phone consultation* OR telephone OR telemental* OR teletherapy OR video call OR televideo OR telehealth OR telemedicine	Text word
2	*satisfaction OR utilization OR engagement OR usage OR adherence OR patient satisfaction OR patient engagement*	All fields
3	predict* OR determin* OR associat* OR correlat*	All fields
4	#1 *AND* #2 *AND* #3	Publication years: 2020-2022 Language: English and German Species: humans

### Selection Process

In August 2022, all the results from the different databases were imported to EndNote (Clarivate), where duplicates were removed. For the next step, 2 reviewers (AN and JB) independently screened the titles and abstracts of the studies, followed by a full-text screening (Cohen κ=0.61). The Rayyan web application was used to support the double-screening process [38]. Disagreements (15/144, 10.4% of studies) were resolved via discussion and consultation with a third reviewer (AH) when needed.

### Data Collection Process

Relevant data from articles that passed the full-text screening were extracted by 1 reviewer (JB) and crosschecked by a second reviewer (AN) using an Excel spreadsheet (Microsoft Corp). The information that was extracted included study characteristics (author, year, study design, country, study period, and data source), population characteristics (sample size, sex, and age), setting (psychiatric care setting and telemental health service type), outcome definition, determinants, analytic approach, and key findings. For missing information or for reasons of clarification, the corresponding authors of the studies were contacted.

### Quality Assessment

The risk of bias was assessed by 2 reviewers independently (AN and JB) using the assessment tool for observational cohort and cross-sectional studies by the National Heart, Lung and Blood Institute [39]. Disagreements were resolved via discussion and consultation with a third reviewer (AH) when needed.

### Synthesis Methods

A formal narrative synthesis of the study results was conducted following the current reporting guidelines for syntheses without meta-analysis in systematic reviews [40]. General study characteristics were summarized in a tabular format. Key findings concerning the determinants of patient use and satisfaction were grouped into categories based on the UTAUT constructs. The UTAUT constructs were adapted and extended depending on the focus of the different studies and the pandemic context. The final categories included performance expectancy, effort expectancy, facilitating conditions, and experience. Age and gender were included into a larger category that contained sociodemographic determinants. The social influence category was adapted to include psychosocial influence to account for the special pandemic situation. Owing to the pandemic circumstances, voluntariness of use was excluded as a category because there was often no option to choose between in-person and telemental health visits. In addition, health- and service-related factors were added as categories to account for potential satisfaction-specific determinants. A meta-analysis of the results was not conducted because of the high heterogeneity across the study designs, outcomes, and effect measures. However, regression coefficients, correlations, and odds ratios were reported when available. In addition, if available, related CIs were specified to assess the certainty of the findings.

## Results

### Quality Assessment

The ratings for study quality are summarized in Tables 2 and 3. Most studies were rated as being of either good (n=12) or fair (n=6) quality. The quality criteria that were most commonly not met in the different studies were the reporting of participation rates (20% fulfilled) and sample size justification, power description or variance, and effect estimates (10% fulfilled).

**Table 2:** Quality assessment for the included studies (studies [41-50]).

Criteria	Studies									
	Ainslie et al [41]	Ceniti et al [42]	Chakawa et al [43]	Connolly et al [44]	Guinart et al [45]	Haxhihamza et al [46]	Hutchison et al [47]	Lewis et al [48]	Lohmiller et al [49]	Lynch et al [50]
1. Was the research question or objective in this paper clearly stated?	Yes	Yes	Yes	Yes	No	No	Yes	Yes	Yes	No
2. Was the study population clearly specified and defined?	Yes	Yes	Yes	Yes	Yes	No	Yes	Yes	Yes	Yes
3. Was the participation rate of eligible persons at least 50%?	N/A^a^	NR^b^	NR	N/A	No	CD^c^	No	Yes	NR	No
4. Were all the subjects selected or recruited from the same or similar populations (including the same time period)? Were inclusion and exclusion criteria for being in the study prespecified and applied uniformly to all participants?	Yes	No	Yes	No	Yes	Yes	Yes	Yes	Yes	No
5. Was a sample size justification, power description, or variance and effect estimates provided?	No	No	No	Yes	No	No	No	No	No	No
6. For the analyses in this paper, were the exposures of interest measured prior to the outcomes being measured?	N/A	N/A	N/A	N/A	N/A	N/A	Yes	N/A	N/A	N/A
7. Was the timeframe sufficient so that one could reasonably expect to see an association between exposure and outcome if it existed?	N/A	N/A	N/A	N/A	N/A	N/A	Yes	N/A	N/A	N/A
8. For exposures that can vary in amount or level, did the study examine different levels of the exposure as related to the outcome (eg, categories of exposure or exposure measured as continuous variable)?	Yes	Yes	Yes	Yes	Yes	CD	Yes	Yes	Yes	Yes
9. Were the exposure measures (independent variables) clearly defined, valid, reliable, and implemented consistently across all study participants?	Yes	Yes	Yes	No (patients vs providers)	Yes	No	Yes	Yes	Yes	Yes
10. Was the exposures assessed more than once over time?	Yes (2 waves)	No	Yes (2 waves)	Yes (2 waves)	No	No	Yes (before and after)	No	No	Yes (3 waves)
11. Were the outcome measures (dependent variables) clearly defined, valid, reliable, and implemented consistently across all study participants?	Yes	Yes	Yes	Yes	Yes	Yes	Yes	Yes	Yes	Yes
12. Were the outcome assessors blinded to the exposure status of participants?	N/A	N/A	N/A	N/A	N/A	N/A	N/A	N/A	N/A	N/A
13. Was loss to follow-up after baseline 20% or less?	Yes/	N/A	Yes	Yes	N/A	N/A	Yes	N/A	N/A	Yes
14. Were key potential confounding variables measured and adjusted statistically for their impact on the relationship between exposures and outcomes?	Yes	Yes	Yes	Yes	No	Yes	No	Yes	Yes	Yes
Quality rating	Good	Good	Fair	Good	Fair	Poor	Fair	Good	Good	Fair

^a^N/A: not applicable.

^b^NR: not reported.

^c^CD: cannot determine.

**Table 3:** Quality assessment for the included studies (studies [51-60]).

Criteria	Studies									
	Meininger et al [51]	Michaels et al [52]	Miu et al [53]	Morgan et al [54]	Nesset et al [55]	Severe et al [56]	Sizer et al [57]	Ter Heide et al [58]	Tobin et al [59]	Vakil et al [60]
1. Was the research question or objective in this paper clearly stated?	Yes	Yes	Yes	Yes	Yes	Yes	Yes	Yes	Yes	Yes
2. Was the study population clearly specified and defined?	Yes	Yes	Yes	Yes	Yes	Yes	Yes	Yes	Yes	Yes
3. Was the participation rate of eligible persons at least 50%?	Yes	No	N/A^a^	NR^b^	No	No	Yes	Yes	N/A	N/A
4. Were all the subjects selected or recruited from the same or similar populations (including the same time period)? Were inclusion and exclusion criteria for being in the study prespecified and applied uniformly to all participants?	Yes	Yes	Yes	Yes	Yes	Yes	Yes	Yes	Yes	Yes
5. Was a sample size justification, power description, or variance and effect estimates provided?	No	No	No	Yes	No	No	No	No	No	No
6. For the analyses in this paper, were the exposures of interest measured prior to the outcomes being measured?	N/A	N/A	N/A	N/A	N/A	N/A	N/A	N/A	N/A	N/A
7. Was the timeframe sufficient so that one could reasonably expect to see an association between exposure and outcome if it existed?	N/A	N/A	N/A	N/A	N/A	N/A	N/A	N/A	N/A	N/A
8. For exposures that can vary in amount or level, did the study examine different levels of the exposure as related to the outcome (eg, categories of exposure or exposure measured as continuous variable)?	Yes	Yes	Yes	Yes	Yes	Yes	Yes	Yes	Yes	Yes
9. Were the exposure measures (independent variables) clearly defined, valid, reliable, and implemented consistently across all study participants?	Yes	Yes	Yes	Yes	Yes	Yes	Yes	Yes	Yes	Yes
10. Was the exposures assessed more than once over time?	No	No	No	No	No	No	No	No	Yes (3 waves)	Yes (2 waves)
11. Were the outcome measures (dependent variables) clearly defined, valid, reliable, and implemented consistently across all study participants?	Yes	Yes	Yes	Yes	Yes	Yes	Yes	Yes	Yes	Yes
12. Were the outcome assessors blinded to the exposure status of participants?	N/A	N/A	N/A	N/A	N/A	N/A	N/A	N/A	N/A	N/A
13. Was loss to follow-up after baseline 20% or less?	N/A	N/A	N/A	N/A	N/A	N/A	N/A	N/A	Yes	Yes
14. Were key potential confounding variables measured and adjusted statistically for their impact on the relationship between exposures and outcomes?	No	Yes	Yes	Yes	No	Yes	Yes	Yes	Yes	Yes
Quality rating	Good	Fair	Good	Good	Poor	Fair	Good	Good	Good	Good

^a^N/A: not applicable.

^b^NR: not reported.

### Overview of Included Studies

**Figure 1:**
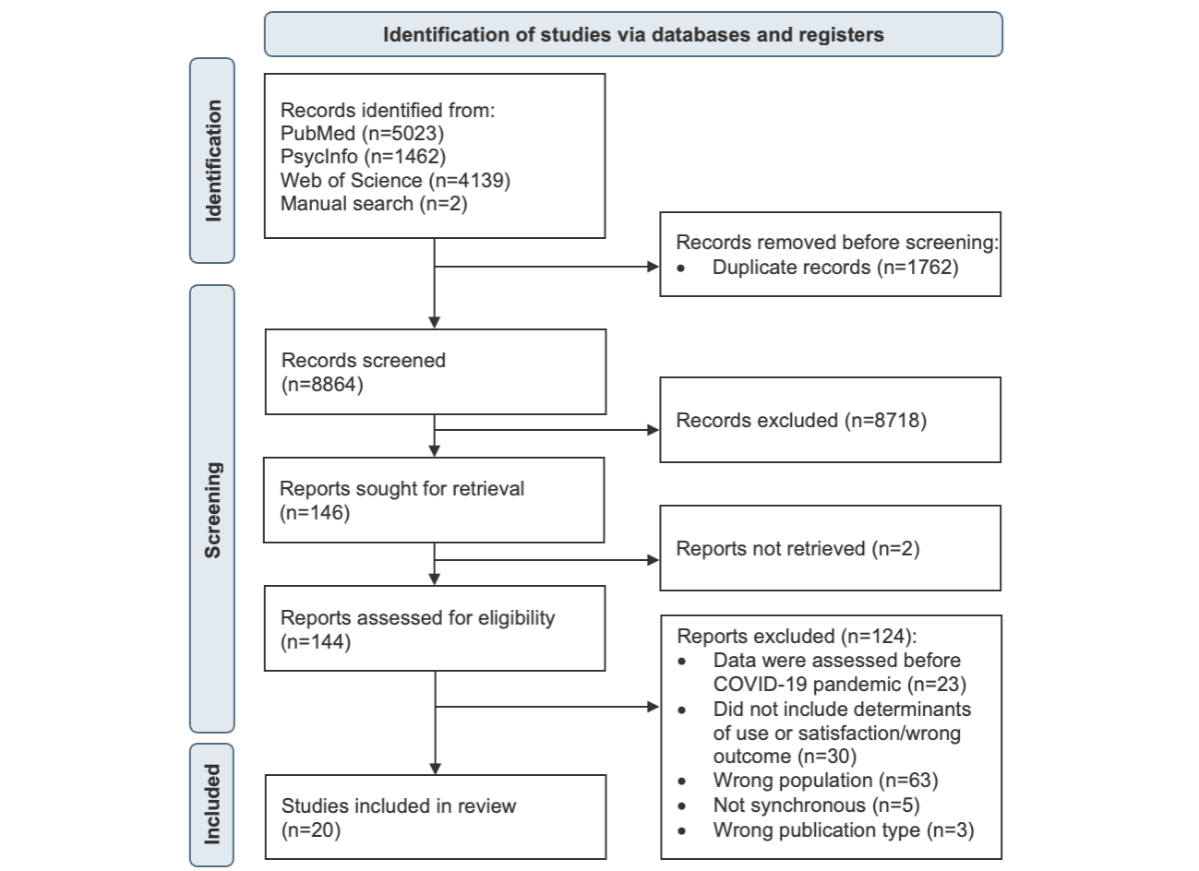
Flow diagram of the study selection process.

After the study selection process, 20 studies remained for the final synthesis (Figure 1; see Multimedia Appendix 1 [41-60] for the citations of all included studies). The main characteristics of these studies are summarized in Tables 4 and 5.

The study samples were predominantly from North America (n=14, with 12 from the United States and 2 from Canada). Furthermore, 5 study samples were from Europe (2 from Germany, 1 from the Netherlands, 1 from Norway, and 1 from North Macedonia), and 1 study sample was from Asia (Israel). Data sources consisted of electronic medical records in 7 studies as well as samples recruited from mental health clinics and community centers in 12 studies. One study used data from a sample that was recruited through targeted emails to mental health organizations nationwide, provincial psychiatric and family physician associations, hospital newsletters, existing participant networks within Canadian Biomarker Integration Network in Depression, and social media. A total of 4 studies were published in 2020, 6 in 2021, 8 in 2022, and 2 in 2023. Although most of the data were collected during the first months of the pandemic, starting from March 2020, some studies also included data from later periods until December 2021.

**Table 4:** Main characteristics of the included studies.

Study, year	Characteristics					
	Study design	Country	Study period	Data source	Population characteristics (sample size; sex: female; age [years], mean [SD])	Psychiatric care setting
Ainslie et al [41], 2022	Observational retrospective study	United States	Study base period: December 1, 2019, to February 29, 2020 Study retention period: April 1 to June 30, 2020 Time trends comparison with study base period: December 1, 2018, to February 28, 2019 Time trends comparison with study retention period: April 1 to June 30, 2019	Electronic medical record data	Medicaid beneficiaries with SMIs^a^, N=15,471 in 2020 Sex: n=6792 (54.7%) Age: mean age not reported	Community mental health centers
Ceniti et al [42], 2022	Cross-sectional, mixed methods study	Canada	October 8, 2020, to February 4, 2021	Recruitment through targeted emails to mental health organizations nationwide, provincial psychiatric and family physician associations, hospital newsletters, existing participant networks within CAN-BIND^b^, and social media	Mental health care users N=332 Sex: n=238 (71.7%) Age: mean age not reported	General remote care experience
Chakawa et al [43], 2021	Comparative study	United States	Before COVID-19: April to October 2019 During COVID-19: April to October 2020	Recruited from clinic	Children aged 1-19 years, N=226 (n=106 for in-person cases before COVID-19, n=120 for telehealth visits cases during COVID-19) Sex: n=83 (36.7%) (before COVID-19 38.7%, during COVID-19 35%) Age: 8.04 (4.30); before COVID-19 mean 7.0, during COVID-19 mean 8.0	Large, inner-city pediatric primary care clinic within a large regional children’s hospital
Connolly et al [44], 2021	Cross-sectional study	United States	Pre-COVID: October 1, 2017, to March 10, 2020 COVID: March 11 to July 10, 2020	Electronic medical record	US veterans with ≥1 mental health outpatient appointment N=2,480,119 before COVID-19; N=1,054,670 during COVID-19; N=954,704 cases from COVID-19 included in pre-COVID cases Sex: before COVID-19 n=325,225 (13.5%), during COVID-19 n=163,186 (15.8%) Age: mean age not reported	Department of Veterans Affairs
Guinart et al [45], 2020	Cross-sectional study	United States	April to June 2020	Recruited from clinics and community centers	Patients using telepsychiatry N=3052 Sex: not reported Age: mean age not reported	18 hospitals and community centers located in rural, suburban, small urban, and large urban areas
Haxhihamza et al [46], 2021	Cross-sectional study	North Macedonia	Not reported	Recruited from clinic	Patients from the ward N=28 Sex: n=11 (37.9%) Age: mean 40.25 (19)	Daily hospital as a part of the University Clinic in Skopje
Hutchison et al [47], 2022	Cross-sectional study	United States	October 2020 to June 2021	Recruited from clinic	Adolescents aged 12-17 years, N=56 Sex: n=37 (66.1%) Age: 14.5 (1.6)	Community mental health clinic
Lewis et al [48], 2021	Cross-sectional study	Israel	Mid-April to mid-May 2020	Recruited from clinic	Eating disorder patients N=63 Sex: n=57 (90.5%) Age: 27.25 (11.47)	Hadarim Eating Disorders Treatment Center (part of the Shavata Mental Health Center)
Lohmiller et al [49], 2021	Cross-sectional study	Germany	July 2020 to February 2021	Recruited from clinic	Patients from the psychosomatic outpatient clinic N=278 Sex: n=182 (83%) Age: 31.5 (range 18-80)	Psychosomatic outpatient clinic at the University Hospital in Tübingen
Lynch et al [50], 2021	Cross-sectional, mixed methods study	United States	February 2 to June 12, 2020 Before COVID-19 (t1): February 2 to March 18, 2020 After COVID-19 1 (t2): March 19 to April 30, 2020 After COVID-19 2 (t3): May 1 to June 12, 2020	Recruited from clinic	Adults with SMI (N=72; t1, n=60; t2, n=64; and t3, n=62) Sex: t1 n=23 (38.3%), t2 n=20 (31.3%), and t3 n=21 (33.9%) Age: t1 28.1 (10), t2 28.22 (10.7), and t3 28.45 (11.14)	Private university-affiliated outpatient psychiatric treatment center
Meininger et al [51], 2022	Cross-sectional study	Germany	July 27 to October 22, 2020	Recruited from clinic	Parents or caregivers answering for or with their children receiving teletherapy N=168 Sex: n=61 (36.3%) Age: 12.29 (4.01)	University Hospital Cologne—School for Child and Adolescent Cognitive Behavior Therapy
Michaels et al [52], 2022	Cross-sectional study	United States	Not reported	Recruited from clinic	College students in a postacute outpatient program who recently required psychiatric hospitalization N=101 Sex: n=72 (74.5%) Age: 22.5 (2.8)	Outpatient mental health clinic at a local psychiatric hospital that provides specialized postacute services to college students
Miu et al [53], 2021	Cross-sectional study	United States	January 16 to April 30, 2020	Electronic medical record	SMI and non-SMI patients N=1444 Sex: n=970 (67.2%) Age: mean age not reported	Outpatient psychiatry clinic of an urban, academic medical center
Morgan et al [54], 2021	Cross-sectional study	United States	March 20 to June 10, 2020	Electronic medical record	Clients in marriage and family training clinics (telehealth sample) N=142 Sex: n=79 (55.6%) Age: 32.56 (16.58)	A total of 2 marriage and family training clinics
Nesset et al [55], 2023	Cross-sectional study	Norway	October-December 2021	Recruited from clinic	Patients from outpatient clinic who attended therapy for aggressive and violent behavior against their partners and children N=28 Sex: n=7 (25%) Age: mean age not reported	Outpatient clinic at St Olav’s University Hospital, Center for Research and Education in Security, Prisons, and Forensic Psychiatry
Severe et al [56], 2020	Cross-sectional study	United States	June-August 2020	Recruited from clinic	Patients who had an in-person appointment date that fell in the first few weeks following the Michigan governor’s stay-at-home edict, necessitating conversion to web-based visits or deferment of in-person care N=244 Sex: n=167 (68.4%) Age: mean age not reported	Outpatient Psychiatry Clinics at the University of Michigan
Sizer et al [57], 2022	Cross-sectional study	United States	April 1, 2020, to March 31, 2021	Electronic medical record	Patients from rural outpatient clinics N=1115 Sex: n=623 (55.9%) Age: not reported	A total of 6 Northeast Delta Human Services Authority outpatient behavioral health clinics
Ter Heide et al [58], 2021	Cross-sectional study	Netherlands	June 3 to July 31, 2020	Recruited from clinic	Patients with complex psychotrauma complaints N=318 Sex: n=130 (40.9%) Age: 52 (11.9)	ARQ Centrum '45 (National institute for diagnostics and treatment of complex psychotrauma complaints)
Tobin et al [59], 2022	Retrospective cohort study	United States	January 1 to December 31, 2020 Before COVID-19: January 1 to March 18, 2020 Telehealth only: March 19 to May 31, 2020, December 1 to December 31, 2020 Choice between telehealth and in-person services: June 1 to November 30, 2020	Electronic medical record	Patients seen by integrated psychology team in general internal medicine N=1075 encounters Sex: n=759 (70.6%) Age: 49.73 (15.89)	Integrated psychology team within the general internal medicine primary care clinic at a large urban health system
Vakil et al [60], 2022	Retrospective cohort study	Canada	Comparison sample: March 19, 2019, to March 18, 2020 COVID-19 sample: March 19, 2020, to April 7, 2021	Electronic medical record	Patients in need of urgent mental health assessment and treatment without referral N=3573 visits Sex: n=1981 (55.4%) Age: 33.9 (13.4)	Crisis Response Center

^a^SMI: serious mental illness.

^b^CAN-BIND: Canadian Biomarker Integration Network in Depression.

**Table 5:** Characteristics of the included studies.

Study, year	Characteristics				
	Telemental health service type (telephone vs video)	Outcome (use vs satisfaction and assessment)	Determinants	Analytic approach	Quality rating
Ainslie et al [41], 2022	All forms of telemental health services	Use: use from pandemic identified by service claim codes; categorized based on percentage of total treatment services during the retention period (low: <25%; medium: 25%-75%; high: >75%)	Sex, age group, diagnosis, and zip code (rural vs urban)	Chi-square test and logistic regression	Good
Ceniti et al [42], 2022	All forms of telemental health services	Use: number of remote visits Satisfaction: 7-point Likert scale (from total dissatisfied to total satisfied) for overall satisfaction with remote care, security, user-friendliness, speed of access and provision of care, continuity of care, convenience, maintenance of therapeutic rapport	Age, type of provider (psychiatrist or family physician vs other mental health care providers), level of connectedness with loved ones, living with others, province or territory, high-risk status for COVID-19, frequency of internet use, and number of people living at home	Chi-square test and Spearman correlation	Good
Chakawa et al [43], 2021	Video (or telephone or audio-only when there were technical problems)	Use: differences in service delivery modality use (in-person visit before COVID-19 vs telehealth use during COVID-19)	Sex, age, referral concern, health insurance type, race or ethnicity, language, controlling for primary care provider, visit control variable (assigned or familiar or not), and appointment type (first or follow-up visit)	Binominal logistic regression	Fair
Connolly et al [44], 2021	Telephone vs video vs in-person services	Use: having had any video experience (before COVID-19 vs during COVID-19); having had ≥50% of visits via phone vs video vs in person	Sex, age, socio economic status, race or ethnicity, rurality, marital status, ≥50% Department of Veterans Affairs disability rating, diagnosis, and history of mental health hospitalization	Binominal and multinomial logistic regression	Good
Guinart et al [45], 2020	Telephone vs video services	Satisfaction: overall experience (telephone or video), perceived helpfulness of remote sessions, challenges and advantages	Age and duration of care	Chi-square test	Fair
Haxhihamza et al [46], 2021	Not specified	Satisfaction: Patient Satisfaction Questionnaire (18 items with 7 dimensions of satisfaction with medical care measured by the Patient Satisfaction Questionnaire-III: general satisfaction, technical quality, interpersonal manner, communication, financial aspects, time spent with doctor, accessibility and convenience)	Age, gender, and place of living	Not specified	Poor
Hutchison et al [47], 2022	Video services	Use: attendance across sessions Satisfaction: Treatment Perception Questionnaire (10 items; general satisfaction and acceptability of mental health services); Internet Evaluation and Utility Questionnaire (15 items; ease of use, convenience, engagement, privacy, satisfaction and acceptability of an internet intervention)	Risk status for adverse mental and behavioral outcomes, and symptom severity	Bivariate correlation and *t* test	Fair
Lewis et al [48], 2021	Web-based platforms, not specified	Satisfaction: Telemedicine Satisfaction Questionnaire (15 items, 5-point Likert scale, 3 factors: quality of care, similarity of remote meetings to face-to-face meetings, perception of the interaction); perspective toward the transition to web-based treatment (6 self-developed statements, 1-5 Likert scale, perception of care, preference of web-based treatment to face-to-face treatment, promotion of this mode of therapy toward others)	Age, gender, education, BMI, duration of treatment in days, past eating disorder, hospitalization, Eating Disorder Examination Questionnaire, Depression, Anxiety and Stress Scales-21, Working Alliance Inventory-S, fear of COVID-19 scale-19S	*t* test and Pearson correlation	Good
Lohmiller et al [49], 2021	Telephone vs video vs in-person services	Satisfaction: self-developed questionnaire with 4 subject areas: patient characterization (10 items), assessment of therapeutic contact (12 items), therapeutic relationship (11 items), hurdles (5 items), 5 additional free-text items	Age, gender, and type of contact	Chi-square test, ANOVA, and hierarchical regression	Good
Lynch et al [50], 2021	Video services	Use: no show or cancellation frequency	Age, gender, race or ethnicity, primary diagnosis, and time period	Model building approach using generalized linear modeling with a Poisson log link (multilevel approach because of nested data structure was used)	Fair
Meininger et al [51], 2022	Video services	Satisfaction: self-developed questionnaire, 11 items: stable internet connection, overall satisfaction, intention to use teletherapy after pandemic=mean satisfaction score; changes in treatment satisfaction and changes in the therapeutic relationship=mean satisfaction change score	Corona Child Stress Scale, psychosocial functioning (Children’s Global Assessment Scale, Child Behavior Checklist [6-18 R] and Youth Self Report [11-18 R]), Checklist for Screening Behavioral and Emotional Problems, and number of teletherapy sessions	Pearson correlation	Good
Michaels et al [52], 2022	Telephone vs video vs in-person services	Satisfaction: preferred telehealth method, overall experience (telephone or video), future telehealth use, perceived helpfulness of remote sessions	Sex, gender, race, and teletherapy format	Chi-square test, Mann-Whitney *U* test, and Kruskal-Wallis test	Fair
Miu et al [53], 2021	Video or telephone vs in-person services	Use: conversion rate to teletherapy for SMI^a^ patients vs non-SMI patients, number of teletherapy sessions between SMI and non-SMI group, differences in new patients starting therapy via telehealth between SMI and non-SMI groups	Age, sex, ethnicity, previous engagement, and SMI vs non-SMI groups	Chi-square test and *t* test	Good
Morgan et al [54], 2021	Video and telephone services	Use: conversion to teletherapy (attendance of at least 1 teletherapy session vs opting out), engagement in teletherapy (number of teletherapy sessions)	Age, gender, race, ethnicity, relationship status, income, education, number of sessions before teletherapy, and case constellation (individual vs relational therapy)	*t* test, logistic regression, and multiple linear regression	Good
Nesset et al [55], 2023	Video services	Satisfaction: Client Satisfaction Questionnaire-8 (8 items measure respondents’ perception of treatment quality)	Gender	*t* test	Poor
Severe et al [56], 2020	Video and telephone services	Use: visit type	Age, sex, race, health insurance type, and number of previous clinic visits	Multiple logistic regression	Fair
Sizer et al [57], 2022	Video and telephone services	Use: number of visits	Age, gender, education (number of school years), race, referral source, monthly income, discharge, chronic condition, number of diagnoses, primary diagnosis type	Negative binomial regression	Good
Ter Heide et al [58], 2021	Video services	Use: 1 item: how did you stay in touch with your therapist during the past 2 mo? (Multiple answers could be given: face-to-face, via videoconferencing, via telephone, through email or chat, not at all) Satisfaction: one item: how satisfied were you with this form of contact, rated on a scale from 0 (not at all satisfied) to 10 (as satisfied as can be)?	Age, gender, level of education, refugee status, Brief Symptom Inventory, Cantril Ladder (life satisfaction), COVID-19 stress level	Pearson product-moment correlation, MANCOVA^b^, ANCOVA^c^, chi-square test, binary logistic regression, and *t* test (2-tailed)	Good
Tobin et al [59], 2023	Telephone vs video vs in-person services	Use: visit type	Age, sex, race, and health insurance type	Logistic regression	Good
Vakil et al [60], 2022	Video or telephone vs in-person services	Use: visit type	Age, sex, distance to crisis response center, household income, prior visit to the center within 1 year, suicidal behavior, diagnosis, visit characteristics (day of the week, time of day, and period of pandemic)	Binary logistic regression	Good

^a^SMI: serious mental illness.

^b^MANCOVA: multivariate analysis of covariance.

^c^ANCOVA: analysis of covariance.

Patient use was examined in 10 studies [41,43,44,50,53,54,56,57,59,60], patient satisfaction in 7 studies [45,46,48,49,51,52,55], and both outcomes were observed in 3 studies [42,47,58]. Patient use was mostly defined as having at least 1 telemental health visit during the pandemic [43,44,53,54,56,58-60]. However, others have also considered the number of telemental health visits [42,53,54,57] and the percentage of telemental health services in overall mental health service use during the pandemic [41,44] or attendance [47,50]. For patient satisfaction, 6 studies used self-developed items and scales [42,45,49,51,52,58], whereas 4 studies used established instruments (ie, Telemedicine Satisfaction Questionnaire [61], Client Satisfaction Questionnaire [62], Patient Satisfaction Questionnaire [63], Treatment Perception Questionnaire [64], and Internet Evaluation and Utility Questionnaire [65]) [46-48,55]. The satisfaction questionnaires mainly focused on the overall satisfaction with the services. Nevertheless, specific satisfaction areas such as satisfaction with the therapeutic relationship and interaction, quality of care, technical aspects, or utility were also addressed.

Most samples included adult populations [42,44,46,49,50,52,55,57,58]. However, children or adolescents were also considered in other studies [41,45,48,53,54,56]. Moreover, some studies exclusively used data collected from children and adolescents [43,47,51]. The sample sizes ranged from 28 to 1,054,670 individuals, with 5 studies including less than 100 individuals, 8 including more than 100 individuals, and 7 including more than 1000 individuals. The proportion of female participants ranged from 15.8% (Department of Veterans Affairs [44]) to 90.5% (patients with an eating disorder [48]). The mean percentage of female participants in the included studies was approximately 55%.

Although none of the included studies used a theoretical model as a background for their analysis, the following sections are based on the UTAUT dimensions to allow for some theoretical context. This may guide future research in this area.

### Patient Use

#### Overview

Key findings for the determinants of patient use of telemental health services are summarized in Table 6 (if reported, adjusted results are presented).

**Table 6:** Key findings of the included studies for determinants^a^ of patient use.

Study, year	Sociodemographic factors (eg, sex, age, race, education, and area lived in)	Health factors (eg, diagnosis, symptoms, and symptom severity)	Service factors (eg, video or telephone and duration of treatment)	Experience (with mental health services)	Facilitating conditions (eg, electronic devices, internet connection, and insurance)
Ainslie et al [41], 2022	Female sex was negatively associated with going from low to either moderate or high telemedicine use (OR^b^ 0.87, 95% CI 0.86-0.92). Compared with patients aged ≥55 y, patients aged 0-12 y (OR 1.18, 95% CI 1.09-1.27) and 13-17 y (OR 1.16, 95% CI 1.09-1.25) had greater odds; patients aged 18-34 y (OR 0.74, 95% CI 0.70-0.79) and 35-54 y (OR 0.79, 95% CI 0.74-0.84) had lower odds of progressing from low to either moderate or high telemedicine use. Living in an urban or rural area did not significantly change the probability for telemedicine use (*P*=.009).	Except for bipolar disorder (OR 0.93, 95% CI 0.84-1.02), patients with diagnoses other than schizophrenia (reference) were negatively associated with progressing from low to either moderate or high use (major depression, OR 0.73, 95% CI 0.68-0.78; PTSD^c^, OR 0.77, 95% CI 0.72-0.83; and anxiety or other disorders, OR 0.69, 95% CI 0.65-0.74).	—^d^	—	—
Ceniti et al [42], 2022	There were no significant age differences (≥50 vs <50 y) regarding video vs telephone service use (detailed results, including numbers, were not reported).	—	—	—	—
Chakawa et al [43], 2021	Black children were less likely to have telemental health visits than White children compared with in-person visits (OR 0.35, 95% CI 0.16-0.76, *P*=.008). Hispanic (reference White: OR 0.45, 95% CI 0.17-1.19, *P*=.11) or other race or ethnicity (reference White: OR 0.57, 95% CI 0.21-1.53, *P*=.26) were not significantly associated with telemental health service use compared with in-person visits. Telemental health service use was not significantly associated with sex (OR 0.64, 95% CI 0.33-1.23, *P*=.18), age (OR 1.22, 95% CI 0.62-2.51, *P*=.54), and language (OR 0.69, 95% CI 0.25-1.89, *P*=.47) compared with in-person visits.	Children with internalizing problems were more likely to have telemental health visits than children with externalizing problems compared with in-person visits (OR 2.78, 95% CI 1.19-6.45, *P*=.02). Other primary referral concerns were not significantly associated with telemental health service use compared with in-person visits (reference; externalizing: OR 1.24, 95% CI 0.57-2.71, *P*=.59).	—	—	Telemental health service use was not significantly associated with health insurance type compared with in-person visits (OR 1.68, 95% CI 0.74-3.82, *P*=.22).
Connolly et al [44], 2022	Female sex was associated with having at least 1 video visit (OR 1.46, 95% CI 1.44-1.48, *P*<.01), having ≥50% of visits via video vs in-person (OR 1.64, 95% CI 1.60-1.68, *P*<.01), having ≥50% of visits via phone vs in-person (OR 1.17, 95% CI 1.15-1.19, *P*<.01), and having ≥50% of visits via video vs phone (OR 1.41, 95% CI 1.38-1.43, *P*<.01). Age was negatively associated with having at least 1 video visit (ORs ranged from 0.27 to 0.92, *P*<.01), having ≥50% of visits via video vs in-person (ORs ranged from 0.23 to 0.91, *P*<.01), having ≥50% of visits via phone vs in-person (ORs ranged from 0.69 to 1.04, *P*<.01), and having ≥50% of visits via video vs phones (ORs ranged from 0.33 to 0.94, *P*<.01). Race and ethnicity was associated with having at least 1 video visit (reference White and non-Hispanic: Black and non-Hispanic OR 0.97, 95% CI 0.96-0.98, *P*<.01; other race and non-Hispanic OR 1.21, 95% CI 1.18-1.25, *P*<.01; Hispanic OR 1.16, 95% CI 1.14-1.18, *P*<.01), having ≥50% of visits via video vs in-person (reference White and non-Hispanic: Black and non-Hispanic OR 0.86, 95% CI 0.84-0.88, *P*<.01; other race and non-Hispanic OR 1.18, 95% CI 1.13-1.23, *P*<.01; Hispanic OR 1.09, 95% CI 1.06-1.12, *P*<.01), having ≥50% of visits via phone vs in-person (reference White and non-Hispanic: Black and non-Hispanic, OR 0.88, 95% CI 0.87-0.89, *P*<.01; other race and non-Hispanic, OR 0.93, 95% CI 0.90-0.96, *P*<.01; Hispanic, OR 0.95, 95% CI 0.93-0.97, *P*<.01), and having ≥50% of visits via video vs phone (reference White and non-Hispanic: Black and non-Hispanic, OR 0.97, 95% CI 0.96-0.99, *P*<.01; other race and non-Hispanic, OR 1.27, 95% CI 1.23-1.31, *P*<.01; Hispanic, OR 1.15, 95% CI 1.13-1.18, *P*<.01). A low socioeconomic status (most disadvantaged tercile) was negatively associated with having at least 1 video visit (OR 0.68, 95% CI 0.67-0.69, *P*<.01), having ≥50% of visits via video vs in-person (OR 0.62, 95% CI 0.60-0.63, *P*<.01), having ≥50% of visits via phone vs in-person (OR 0.96, 95% CI 0.94-0.97, *P*<.01), and having ≥50% of visits via video vs phone (OR 0.64, 95% CI 0.63-0.65, *P*<.01). Rurality was partly associated with having at least 1 video visit (reference urban: rural OR 1.14, 95% CI 1.12-1.16, *P*<.01; highly rural OR 1.22, 95% CI 1.14-1.31, *P*<.01), having ≥50% of visits via video vs in-person (reference urban: rural, OR 1.00, 95% CI 0.98-1.02, *P*>.05; highly rural, OR 1.24, 95% CI 1.13-1.36, *P*<.01), having ≥50% of visits via phone vs in-person (reference urban: rural, OR 1.14, 95% CI 1.12-1.16, *P*<.01; highly rural, OR 1.22, 95% CI 1.14-1.31, *P*<.01), and having ≥50% of visits via video vs phone (reference urban: rural, OR 0.88, 95% CI 0.86-0.89, *P*<.01; highly rural, OR 1.01, 95% CI 0.94-1.09, *P*>.05). Not being married and being divorced, separated, or widowed compared with being married was negatively associated with having at least 1 video visit (ORs ranged from 0.92 to 0.93, *P*<.01), having ≥50% of visits via video vs in-person (ORs ranged from 0.82 to 0.83, *P*<.01), having ≥50% of visits via phone vs in-person (ORs ranged from 0.91 to 0.93, *P*<.01), and having ≥50% of visits via video vs phones (ORs ranged from 0.89 to 0.90, *P*<.01).	Schizophrenia diagnosis was negatively associated with having at least 1 video visit (OR 0.69, 95% CI 0.67-0.71, *P*<.01), having ≥50% of visits via video vs in-person (OR 0.36, 95% CI 0.34-0.37, *P*<.01), having ≥50% of visits via phone vs in-person (OR 0.64, 95% CI 0.62-0.65, *P*<.01), and having ≥50% of visits via video vs phone (OR 0.56, 95% CI 0.54-0.59, *P*<.01). Depression diagnosis was associated with having at least 1 video visit (OR 1.06, 95% CI 1.05-1.07, *P*<.01), having ≥50% of visits via video vs in-person (OR 1.10, 95% CI 1.08-1.12, *P*<.01), and having ≥50% of visits via phone vs in-person (OR 1.10, 95% CI 1.09-1.12, *P*<.01). It was not significantly associated with having ≥50% of visits via video vs phone (OR 1.00, 95% CI 0.99-1.02, *P*>.05). Anxiety disorder diagnosis was associated with having at least 1 video visit (OR 1.03, 95% CI 1.02-1.04, *P*<.01), having ≥50% of visits via video vs in-person (OR 1.03, 95% CI 1.02-1.05, *P*<.01), and having ≥50% of visits via phone vs in-person (OR 1.04, 95% CI 1.03-1.06, *P*<.01). It was not significantly associated with having ≥50% of visits via video vs phone (OR 1.00, 95% CI 0.98-1.01, *P*>.05). Bipolar disorder diagnosis was negatively associated with having ≥50% of visits via video vs in-person (OR 0.89, 95% CI 0.86-0.91, *P*<.01) and having ≥50% of visits via video vs phone (OR 0.89, 95% CI 0.86-0.91, *P*<.01). It was not significantly associated with having at least 1 video visit (OR 1.00, 95% CI 0.98-1.02, *P*>.05) and having ≥50% of visits via phone vs in-person (OR 1.00, 95% CI 0.98-1.02, *P*>.05). PTSD diagnosis was associated with having ≥50% of visits via video vs in-person (OR 1.11, 95% CI 1.09-1.13, *P*<.01), having ≥50% of visits via phone vs in-person (OR 1.16, 95% CI 1.15-1.18, *P*<.01), and negatively with having ≥50% of visits via video vs phone (OR 0.96, 95% CI 0.94-0.97, *P*<.01). It was not significantly associated with having at least 1 video visit (OR 1.01, 95% CI 0.98-1.01, *P*>.05). Substance use disorder diagnosis was negatively associated with having ≥50% of visits via video vs in-person (OR 0.75, 95% CI 0.73-0.76, *P*<.01), having ≥50% of visits via phone vs in-person (OR 0.87, 95% CI 0.86-0.89, *P*<.01), and having ≥50% of visits via video vs phone (OR 0.86, 95% CI 0.84-0.87, *P*<.01). It was not significantly associated with having at least 1 video visit (OR 1.00, 95% CI 0.98-1.01, *P*>.05). Past mental health hospitalization was associated with having at least one video visit (OR 1.09, 95% CI 1.07-1.12, *P*<.01) and negatively associated with having ≥50% of visits via video vs in-person (OR 0.56, 95% CI 0.54-0.58, *P*<.01), having ≥50% of visits via phone vs in-person (OR 0.62, 95% CI 0.61-0.64, *P*<.01) and having ≥50% of visits via video vs phone (OR 0.88, 95% CI 0.86-0.91, *P*<.01). A disability rating of ≥50% was associated with having at least 1 video visit (OR 1.05, 95% CI 1.03-1.06, *P*<.01), having ≥50% of visits via video vs in-person (OR 1.07, 95% CI 1.05-1.10, *P*<.01), having ≥50% of visits via phone vs in-person (OR 1.02, 95% CI 1.00-1.04, *P*<.01), and having ≥50% of visits via video vs phone (OR 1.05, 95% CI 1.03-1.07, *P*<.01).	—	—	—
Hutchison et al [47], 2022	—	Increased baseline depression symptomology (r=−0.34, *P*<.05) and baseline anxiety symptomology (r=−0.32, *P*<.05) were associated with lower internet intervention use. Baseline somatic symptoms were not significantly correlated with internet intervention use (r=−0.26, *P*>.05). No significant differences in attendance or retention rate were found for the moderate-risk and high-risk group (t=1.22, *P*=.23; t=0.20, *P*=.84).	—	—	—
Lynch et al [50], 2021	No significant associations with number of missed or cancelled sessions for age, gender, and race or ethnicity were found (detailed results, including numbers, were not reported).	Having had at least 1 psychotic episode was associated with fewer missed or cancelled sessions (B=−0.49, *P*<.05).	—	The mean no show or cancellation rate was 37% less during time 3 (post 2, week 13-18) compared with no show or cancellations while sessions were held in person (B=−0.47, *P*<.05).	—
Miu et al [53], 2021	Older age was significantly associated with a smaller likelihood for conversion to teletherapy (B=−0.010, *P*=.01, OR 0.99, 95% CI 0.98-0.99). The SMI^e^ status × age interaction was nonsignificant (B=0.021, *P*=.13, OR 1.02), meaning that the conversion for SMI and non-SMI groups did not depend on age. Nonsignificant predictors for conversion to teletherapy were sex (B=0.229, *P*=.13, OR 1.26, 95% CI 0.94-1.69) and ethnicity (reference non-Hispanic or Latino: Hispanic or Latino, B=−0.170, *P*=.44, OR 0.84, 95% CI 0.55-1.30; other, B=0.150, *P*=.56, OR 1.16, 95% CI 0.70-1.93).	SMI status did not significantly predict conversion to telehealth (B=0.095, *P*=.63, OR 1.10, 95% CI 0.75-1.62). The proportion of new patients starting teletherapy did not significantly differ by SMI status (χ21=1.2, *P*=.27). Patients with SMI had significantly higher numbers of telehealth visits compared with the non-SMI group (SMI: mean 1.47, SD 2.01; non-SMI: mean 1.04, SD 1.42; t251,154=−3.027, *P*=.003).	—	Patients’ previous engagement was not significantly associated with conversion to teletherapy (B=0.003, *P*=.41, OR 1.00, 95% CI 0.99-1.01). The SMI status × previous engagement interaction was nonsignificant (B=0.007, *P*=.43, OR 1.00), meaning that conversion for SMI and non-SMI groups did not depend on patients’ previous engagement.	—
Morgan et al [54], 2021	Conversion to teletherapy was significantly associated with Hispanic ethnicity (χ21=6.7, *P*=.01, also in logistic regression model: B=2.425, *P*<.05, OR 11.30). The association between conversion to teletherapy and the following demographic characteristics were not significant: age (t165=−1.474, *P*=.74), gender (χ21=2.1, *P*=.15), being a person of color (χ21=3.2, *P*=.07), poverty (χ21=3.0, *P*=.09), low educational attainment (χ21=0.1, *P*=.80), and household poverty status (χ21=1.2, *P*=.27). Engagement in teletherapy was not significantly associated with ethnicity (B=1.15, β=.125, *P*>.05).	—	Clients in individual therapy (individual vs relational case constellations) were more likely to convert to teletherapy (χ21=4.2, *P*=.04), also in logistic regression model (B=−1.38, *P*<.05, OR 0.25). Engagement in teletherapy was associated with individual therapy (B=−2.34, *P*<.001, β=−.289).	The number of sessions attended before the conversion to teletherapy was not significantly associated with the conversion to teletherapy (B=0.01, *P*>.05, OR 1.01) and engagement in teletherapy (B=0.02, *P*<.05, β=.179).	—
Severe et al [56], 2020	Patient age was associated with the initial choice in visit type (*P*<.001). Patients aged ≥44 y were more likely than patients aged <44 y to choose telephone visits (RRR^f^=1.2; 95% CI 1.06-1.35). Sex (*P*=.99) and race (*P*=.06) were not significantly associated with the initial choice in visit type (delineating new and preexisting patients).	—	—	The number of previous clinic visits was not significantly associated with the initial choice in visit type (delineating new and preexisting patients; *P*=.63).	Health insurance type was not significantly associated with the initial choice in visit type (delineating new and preexisting patients; *P*=.08).
Sizer et al [57], 2022	Female sex was associated with an increased number of telehealth visits (reference male: IRR^g^=1.11, *P*<.05). Age was negatively associated with the number of telehealth visits (reference>60 years: 18-30 y IRR=1.16, *P*<.10; 31-45 y IRR=1.22, *P*<.01; 46-60 y IRR=1.22, *P*<.01). The number of school years was positively associated with the number of telehealth visits (IRR=1.01, *P*<.05). No significant associations were found for race (IRR ranged from 0.74 to 0.99, *P*>.10) and monthly income (IRR=1.03, *P*>.10).	The number of telehealth visits among patients with schizophrenia spectrum and other psychotic disorders decreased by 15% compared with patients with depressive disorders (IRR=0.85, *P*<.01). No significant results were found for other primary diagnosis types (IRR ranged from 0.903 to 0.959, *P*>.10). The number of diagnosed mental illnesses was positively associated with the number of telehealth visits (IRR=1.07, *P*<.01). The presence of other chronic health conditions was positively associated with the number of telehealth visits (IRR=1.10, *P*<.05).	Discharge from clinic was negatively associated with the number of telehealth visits (IRR=0.55, *P*<.01). No significant associations were found for referral source (self vs external source) and the number of telehealth visits (IRR=1.00, *P*>.10).	—	—
Ter Heide et al [58], 2021	Refugee status was negatively associated with VCT^h^ use (B=1.35, *P*<.01, OR 3.86, 95% CI 1.80-8.28).	General psychopathology was negatively associated with VCT use (B=−0.58, *P*<.01, OR 0.56, 95% CI 0.39-0.56).	—	—	—
Tobin et al [59], 2023	Older (OR 1.04, *P*<.001) and Black patients compared with White patients (OR 3.85, *P*<.05) were more likely to complete audio-only visits compared with video visits when only telehealth visits were offered (n=359). Gender was not significantly associated with telehealth visit type during that period (OR 1.04, *P*=.90). No significant associations with demographic predictors were found (age, gender, and race; ORs ranged from 0.49 to 1.60, *P* value ranged from .07 to .25) when in-person and telehealth visits were offered (n=222).	—	—	—	Patients with Medicare (OR 3.46, *P*<.001) and Medicaid (OR 3.43, *P*<.001) health insurance compared with private payers were more likely to complete audio-only visits than video visits when only telehealth visits were offered (n=359). Health insurance type was not significantly associated with use of telehealth visits when in-person and telehealth visits were offered (n=222; reference private payer: Medicare, OR 2.01, *P*=.10; Medicaid, OR 1.07, *P*=.83).
Vakil et al [60], 2022	Male sex was negatively associated with telehealth visit use (reference female: male, OR 0.76, 95% CI 0.64-0.91, *P*=.002; other, OR 0.43, 95% CI 0.13-1.45, *P*=.18). Older age was positively associated with telehealth visit use (OR 1.01, 95% CI 1.00-1.01, *P*=.03). Patients with income Q2^i^ were more likely to use telehealth visits compared with the lowest income group Q1 (reference Q1: Q2, OR 1.32, 95% CI 1.01-1.74, *P*=.046). Other Qs did not significantly differ from the lowest income group Q1 concerning telehealth visit use (ORs ranged from 0.94 to 1.29, *P* values ranged from .10 to .69). The distance between the individual’s residence and the clinic was positively associated with telehealth visit use (OR 1.04, 95% CI 1.02-1.07, *P*=.001).	Absence of suicidal behavior (reference none: ideation, OR 0.74, 95% CI 0.61-0.90, *P*=.003; planning, OR 0.55, 95% CI 0.38-0.79, *P*=.001; self-harm or attempt, OR 0.62, 95% CI 0.48-0.81, *P*<.001), substance use (reference none: OR 0.60, 95% CI 0.50-0.72, *P*<.001), psychotic symptoms (reference absent: OR 0.41, 95% CI 0.30-0.56, *P*<.001) and cognitive impairment (reference absent: OR 0.53, 95% CI 0.34-0.84, *P*=.007) were associated with telehealth visit use Presence of personality problems (OR 1.13, 95% CI 0.92-1.40, *P*=.26), depressive or anxiety problems (OR 1.26, 95% CI 0.98-1.63, *P*=.07), bipolar spectrum disorders (OR 0.94, 95% CI 0.65-1.34, *P*=.30), and other mental illnesses (OR 0.82, 95% CI 0.57-1.19, *P*=.30) were not significantly associated with telehealth visit use.	Each pandemic period after the first lockdown (reference lockdown 1: in-between period, OR 0.37, 95% CI 0.23-0.49, *P*<.001; lockdown 2, OR 0.39, 95% CI 0.30-0.52, *P*<.001; after lockdown 2, OR 0.35, 95% CI 0.26-0.49, *P*<.001), overnight visits (reference daytime visits: OR 0.48, 95% CI 0.34-0.67, *P*<.001), and weekend visits (reference weekday visits: OR 0.75, 95% CI 0.61-0.91, *P*=.004) were negatively associated with telehealth visit use.	Patients with a prior visit in the last year were less likely to use telehealth visits (OR 0.75, 95% CI 0.61-0.91, *P*=.004).	—

^a^Psychosocial influence, effort, and performance expectancy were not included as categories in this table because none of the included studies observed the relationship of these determinants with patient use.

^b^OR: odds ratio.

^c^PTSD: posttraumatic stress disorder.

^d^No information present in the study regarding this category of determinants.

^e^SMI: serious mental illness.

^f^RRR: relative risk reduction.

^g^IRR: incidence rate ratio.

^h^VCT: clinical videoconferencing.

^i^Q: income quintile (Q1: lowest and Q5: highest).

#### Sociodemographic Factors

In total, 11 studies examined the relationship between sex and patient use of telemental health services. Approximately half of these studies (n=6) did not find significant sex differences in use [43,50,53,54,56,59]. Nevertheless, 4 studies reported higher use rates in female participants [44,57,58,60]. In contrast, 1 study reported lower odds for female participants to go from low use rates (before the pandemic) to moderate or high use rates during the pandemic [41].

A total of 13 studies examined the relationship between age and patient use of telemental health services. Nearly half of these studies (n=6) found a nonsignificant association of age with patient use [42,43,50,54,58,59]. In contrast, 1 study found that older age was positively associated with telemental health service use [60] and 3 studies found that older patients were more likely to use audio-only formats (eg, telephone services) compared with video formats [44,56,59]. Nevertheless, 3 studies observed a negative association of age with telemental health service use [44,53,57]. Ainslie et al [41] reported mixed findings. In their sample, participants aged 0 to 17 years were more likely than those aged ≥55 years to go from having <25% of mental health services in a remote format (low use) to having 25% to 75% (moderate use) or >75% (high use) of use. However, participants aged 18 to 54 years were less likely than those aged ≥55 years to go from low to moderate or high use.

In total, 8 studies examined the relationship between race or ethnicity and patient use of telemental health services. Of these, 5 studies did not find a significant association [50,53,56,57,59]. However, Tobin et al [59] reported that Black individuals were more likely to use audio-only services, which was also found in the study by Connolly et al [44]. In addition, 2 studies found that Black patients were less likely to use telemental health services and used them less frequently compared with White patients [43,44]. Connolly et al [44] found that other than Black races and Hispanic ethnicity compared with the White race, non-Hispanic race or ethnicity is positively associated with telemental health service use and frequency of video service use (but negatively associated with frequency of phone service use). Although being a person of color was a nonsignificant determinant for the conversion to teletherapy, a relationship between Hispanic ethnicity and the conversion was found in the sample of Morgan et al [54]. However, when examining engagement with teletherapy, no significant association with ethnicity was observed in their sample.

A total of 3 studies examined the relationship of area lived in and patient use of telemental health services. Findings suggested a positive association with rurality: 1 study found that individuals from (highly) rural areas were more likely to use telemental health services [44] and 1 study stated that telehealth users lived further away from the clinic [60]; however, 1 study found no significant association [41].

Other sociodemographic determinants of patient use were considered in very few studies. A low socioeconomic and financial status was associated with lower use in 2 studies [44,60] but failed to significantly predict telemental health service use in 2 other studies [54,57]. Years of schooling were positively associated with the number of visits in the sample by Sizer et al [57]; however, Morgan et al [54] did not find a significant association between educational attainment and opting out of teletherapy after clinical conversion from in-person therapy to teletherapy. In addition, being married was positively associated with telemental health service use and use frequency in 1 study [44]. Language was not significantly associated with use, and refugee status was associated with lower odds of telemental health use in single studies [43,58].

#### Health Factors

A total of 9 studies examined the relationship of psychological symptom severity or diagnosis and patient use of telemental health services. Most of these studies (n=5) found that individuals with higher symptom severity (eg, patients with schizophrenia) had lower use rates [44,47,57,58,60]. However, the number of diagnoses, depression, anxiety or posttraumatic stress disorder diagnosis, past psychotic episodes, and serious mental illness status were each associated with a higher use frequency or fewer missed sessions in single studies [44,50,53,57]. Similarly, Ainslie et al [41] reported that individuals with schizophrenia were more likely to go from low to moderate or high use than individuals with other diagnoses. Nevertheless, the risk status for adverse mental and behavioral outcomes and serious mental illness status were not significantly associated with use and visit intensity in single studies [47,53]. In addition, Chakawa et al [43] found that children with internalizing problems were more likely to have a telemental health visit than children with externalizing problems.

Furthermore, the presence of chronic health conditions was associated with a higher number of visits in the sample studied by Sizer et al [57]. A disability rating of ≥50% in US veterans was positively associated with telemental health service use and frequency of use in 1 study [44].

#### Service Factors

A total of 3 studies examined the relationship between service factors and patient use of telemental health services. Morgan et al [54] found that patients undergoing individual therapy were more likely to convert to telemental health services. Referral source (self vs external sources) was not significantly associated with use rates [57]. Regarding service times, Vakil et al [60] stated that telehealth visits were significantly less likely during each pandemic period after the first lockdown, for nighttime visits (compared with daytime visits) and weekend visits (compared with weekday visits).

#### Experience

A total of 5 studies examined the relationship between experience with telemental health services and patient use of telemental health services. Previous engagement in mental health services was found to be negatively associated with telehealth visit use in the sample studied by Vakil et al [60] but failed to predict use in 2 other studies [53,56]. Although the number of sessions attended before teletherapy was not significantly associated with conversion to teletherapy in the analysis by Morgan et al [54], it was found to significantly predict the number of telemental health visits in this sample. Moreover, Lynch et al [50] reported that longer duration of participation in telemental health services was associated with fewer missed sessions.

#### Facilitating Conditions

A total of 3 studies examined the relationship between facilitating conditions and patient use of telemental health services. Health insurance type was not significantly associated with patient use in these studies [43,56,59]. Nevertheless, Tobin et al [59] reported that Medicare- or Medicaid-insured individuals used audio-only formats more often than private payers.

#### Psychosocial Influence, Effort and Performance Expectancy

None of the included studies examined the relationship between psychosocial factors, effort or performance expectancy and patient use of telemental health services.

### Patient Satisfaction

#### Overview

Key findings for the determinants of patient satisfaction with telemental health services are summarized in Table 7 (if reported, adjusted results are presented).

**Table 7:** Key findings of the included studies for determinants^a^ of patient satisfaction.

Study, year	Sociodemographic factors (eg, sex, age, race, education, and area lived in)	Health factors (eg, diagnosis, symptoms, and symptom severity)	Service factors (eg, video or telephone and duration of treatment)	Experience (with mental health services)	Psychosocial influence (what do families and peers think about program or psychosocial impact)	Facilitating conditions (eg, electronic devices, internet connection, and insurance)
Ceniti et al [42], 2022	Satisfaction was not significantly associated with age (≥50 y vs <50 y) and number of people living in the household (detailed results, including numbers, were not reported). Living with others was significantly associated with satisfaction (χ21=5.8, *P*=.02). Satisfaction was greater in users from Ontario compared with those from other Canadian provinces (χ21=3.9, *P*=.047).	Satisfaction was not significantly associated with high-risk status for COVID-19 (detailed results, including numbers, were not reported).	Video services (compared with telephone) were associated with greater satisfaction (User-MD^b^ χ21=6.1, *P*=.01; User-HCP^c^ χ21=6.6, *P*=.01). No significant differences between user-groups (psychiatrists or family physicians vs other mental health care providers) in overall satisfaction were found (detailed results, including numbers, were not reported).	—^d^	Level of connectedness with loved ones was positively correlated with overall remote care satisfaction (*r*=.197, *P*=.007) and satisfaction with therapeutic rapport (*r*=.155, *P*=.03).	Satisfaction was not significantly associated with frequency of internet use (detailed results, including numbers, were not reported).
Guinart et al [45], 2020	Significant age differences for telephone services were found (χ224=46.3, *P*=.004). A lower proportion of patients aged 55-64 y described their experience as excellent compared with other age groups (χ24=12.8, *P*=.01). A higher proportion of patients aged 45-54 y rated their experience as poor compared with other age groups (χ24=10.5, *P*=.03).	—	—	Patients under care for <1 y endorsed missing the clinic and feeling connected to it less frequently than other groups (*χ*^2^_6_=21.5, *P*=.002).	—	—
Haxhihamza et al [46], 2021	Satisfaction was not significantly associated with gender, age, and place of living (detailed results, including numbers, were not reported).	—	—	—	—	—
Hutchison et al [47], 2022	—	None of the baseline psychological symptoms were correlated with treatment satisfaction (r values ranged between −0.13 and 0.01, *P*>.05). Adolescents in the moderate-risk group reported significantly higher satisfaction with the intervention than those in the high-risk group (t=2.03, *P*<.05, Cohen d=0.60).	—	—	—	—
Lewis et al [48], 2021	No significant correlations with the views toward the transition to web-based therapy for age (r=.036, *P*=.78), gender (r=.006, *P*=.96), and education (r=.092, *P*=.47) were found. No significant correlations with the TSQ^e^ for age (similarity scale: r=.182, *P*=.15; quality scale: r=−.047, *P*=.72), gender (similarity scale: r=.067, *P*=.60; quality scale: r=.146, *P*=.25), and education (similarity scale: r=.093, *P*=.47; quality scale: r=−.017, *P*=.89) were found.	No significant correlations with the views toward the transition to web-based therapy for past eating disorder hospitalization (t=0.152, *P*=.24), EDE-Q^f^ scales (r values ranged from −0.168 to −0.094, *P* values ranged from .19 to .47) and Depression, Anxiety and Stress Scales-21 scales (r values ranged from −0.162 to −0.080, *P* values ranged from .21 to .53) were observed. No significant associations of the TSQ with past eating disorder hospitalization (similarity scale: t=0.149, *P*=.24; quality scale: t=0.061, *P*=.63), EDE-Q scales (r values ranged from −0.100 to 0.101, *P* values ranged from .43 to .77) and Depression, Anxiety and Stress Scales-21 scales (r values ranged from −0.121 to 0.094, *P* values ranged from .34 to .84) were found. TSQ scores did not significantly differ between eating disorder diagnoses (detailed results, including numbers, were not reported). No significant correlation of the views toward the transition to web-based therapy with BMI (r=0.226, *P*=.08) were found. BMI was not significantly correlated with the TSQ (similarity scale: r=0.221, *P*=.09; quality scale: r=−0.011, *P*=.93).	—	Treatment duration correlated with positive views toward the transition to online therapy (r=0.291, *P*=.02). Treatment duration was not significantly correlated with the TSQ (similarity scale: r=0.124, *P*=.34; quality scale: r=−0.144, *P*=.26).	The fear of COVID-19 scale-19S score correlated with positive views toward the transition to web-based therapy for (r=0.276, *P*=.03). The fear of COVID-19 scale-19S score was not significantly associated with the TSQ (similarity scale: r=−0.193, *P*=.13; quality scale: r=−0.143, *P*=.26).	—
Lohmiller et al [49], 2021	No significant associations of age (F1,277=0.18, *P*=.67) and gender (detailed results, including numbers, were not reported) with the overall “assessment of therapeutic contact” were found. However, a significant association of age with the item “assessment of therapeutic contact as personal” was found (F1,277=4.50, *P*=.04) indicating that older individuals perceived the video format as partly more impersonal. When looking at “hurdles” age was significantly associated with single items: “The necessary technology/framework conditions overwhelmed me” (F1,277=7.85, *P*=.005) indicating that older individuals perceived the video format as more challenging and “I was able to fully concentrate on the content of the conversation” (F1,277=14.85, *P*<.001) indicating that older individuals perceived the video format to be more impersonal and depersonalized.	—	Significant differences were found in the items: global judgment conversation contact (F2,275=3.39, *P*=.04), pleasantness (F2,275=3.35, *P*=.04), friendliness (F2,275=5.55, *P*=.004), and feeling comfortable (F2, 275=8.49, *P*<.001), all favoring video consultations compared with phone and office consultation. The other items of the “assessment of therapeutic contact” showed no significant differences (P≥.05). The assessment of the “therapeutic relationship” did not significantly differ between groups, except for the item “I have recently started to feel better” (F2,275=4.97, *P*=.008), favoring phone and video contacts.	—	—	—
Meininger et al [51], 2022	—	There were no significant correlations between parent-rated treatment satisfaction and the severity of patients’ symptoms, stress, and psychosocial functioning (detailed results, including numbers, were not reported).	—	Treatment duration correlated positively with parent-rated treatment satisfaction (mean satisfaction score: r=.20, *P*<.02).	—	—
Michaels et al [52], 2022	No sex-based differences in the preferred telehealth method (*P*=.67), experiences using telephone (*P*=.92) or video (*P*=.58), whether patients would use telehealth in the future (*P*=.11) and was perceived as helpful as in-person treatment (*P*=.38) were found. No gender-based differences in the preferred telehealth method (*P*=.64), experiences using telephone (*P*=.63) or video (*P*=.53), whether patients would use telehealth in the future (*P*=.52) and was perceived as helpful as in-person treatment (*P*=.13) were found. No race-based differences in the preferred telehealth method (*P*=.21), experiences using telephone (*P*=.29) or video (*P*=.99) and whether patients would use telehealth in the future (*P*=.15) were found.	—	No between-group differences in preferences for telehealth methods were found (Н1=0.46, *P*=.49). Most of the college therapy and medication group (63/78, 81%) and college medication–only group (20/23, 87%) reported a strong preference for the video format.	—	—	—
Nesset et al [55], 2023	No significant sex differences were found for Client Satisfaction Questionnaire-8 scores (*P* values ranged from .10 to .57 for the different items), except that female participants were more content with the length of the therapy (item 5: mean 3.86, SD 0.378 vs male, mean 2.90, SD 1.01, *P*=.03).	—	—	—	—	—
Ter Heide et al [58], 2021	There was a significant main effect of gender, with female participants reporting significantly higher VCT^g^ satisfaction than male participants (F1,196=10.60, *P*<.01). No significant associations with VCT satisfaction were found for age, refugee status and level of education (detailed results, including numbers, were not reported).	There was a significant main effect of general psychopathology, with general psychopathology being negatively associated with VCT satisfaction (F1,196=6.61, *P*<.05). Among those who reported using VCT, a small, negative correlation between VCT satisfaction and general psychopathology was found (r=−0.18, *P*<.01, n=221).	There was no significant difference in treatment satisfaction between the VCT group and the non-VCT group (t276=−0.237, *P*=.81, n=278).	—	Among those who reported using VCT, a small, negative correlation between satisfaction and coronavirus stress level was found (r=−0.21, *P*<.01, n=228). A small, positive correlation between VCT satisfaction and life satisfaction was found (r=0.27, *P*<.001, n=228).	—

^a^Effort and performance expectancy were not included as categories in this table because none of the included studies observed a relationship between these determinants and patient satisfaction.

^b^User-MD: mental health care users who saw an MD provider (psychiatrist or family physician).

^c^User-HCP: mental health care users who saw another mental health care provider (eg, psychotherapist).

^d^No information present in the study regarding this category of determinants.

^e^TSQ: telemedicine satisfaction questionnaire.

^f^EDE-Q: Eating Disorder Examination Questionnaire.

^g^VCT: clinical videoconferencing.

#### Sociodemographic Factors

A total of 5 studies examined the relationship between sex and patient satisfaction with telemental health services, and all of them did not find a significant association of sex with the satisfaction scores [46,48,49,52,55].

A total of 6 studies examined the relationship between age and patient satisfaction with telemental health services. Most studies (n=4) did not find a significant association between age and satisfaction [42,46,48,58]. Lohmiller et al [49] also did not find a significant association between age and the overall satisfaction with therapeutic contact. However, older age was associated with lower satisfaction for some items, meaning that older individuals perceived the video intervention as less personal and more challenging and found it harder to fully concentrate on the content of the conversation. Guinart et al [45] found lower satisfaction ratings for telephone services among older patients.

One study observed a nonsignificant relationship between race and patient satisfaction with telemental health services [52].

In total, 2 studies examined the relationship between area lived in and patient satisfaction with telemental health services. While Haxhihamza et al [46] did not find a significant association, Ceniti et al [42] reported greater satisfaction ratings in users from Ontario compared with those in other Canadian provinces.

Other sociodemographic determinants of patient satisfaction were considered in some studies. Educational level was observed in 2 studies and was not significantly associated with satisfaction in these samples [48,58]. In addition, Ter Heide et al [58] reported that refugee status is not significantly associated with satisfaction. Moreover, Ceniti et al [42] included living situation of participants as a potential determinant. While the number of people living in the household was not significantly associated with remote care satisfaction, living with others showed a significant association with this outcome.

#### Health Factors

A total of 4 studies examined the relationship between psychological symptom severity and patient satisfaction with telemental health services. Only 1 study found a significant association between symptom severity and satisfaction. In the sample studied by Hutchison et al [47], patients at moderate risk were more satisfied than patients who were at high risk for adverse mental and behavioral outcomes. However, the other 3 studies did not observe significant relationships [42,48,51].

A total of 2 studies examined the relationship between physical health and patient satisfaction with telemental health services. Nonsignificant relationships were found between BMI and high-risk status for COVID-19, with satisfaction in single studies [42,48].

#### Service Factors

A total of 7 studies examined the relationship between service factors and patient satisfaction with telemental health services. Of these, 3 studies reported that telemental health services delivered via video services were associated with higher patient satisfaction than those delivered via telephone services [42,49,52]. However, Ter Heide et al [58] could not find this relationship. Furthermore, the therapeutic alliance bond was associated with higher satisfaction ratings in 1 study [48]. The provider type (psychiatrists or family physicians vs other mental health care providers) was not significantly associated with patient satisfaction in the study by Ceniti et al [42].

#### Experience

A total of 3 studies examined the relationship between experience with telemental health services and patient satisfaction with telemental health services. In the study by Lewis et al [48], longer treatment duration was associated with higher satisfaction, while Guinart et al [45] observed that patients who were under care for less than a year perceived the transition to telemental health services as less negative (missed the clinic less and did not feel less connected). Moreover, the number of telemental health sessions was associated with higher satisfaction ratings in 1 study [51].

#### Psychosocial Influence

A total of 3 studies examined the relationship between psychosocial factors and patient satisfaction with telemental health services. Level of connectedness with loved ones and life satisfaction were associated with greater patient satisfaction [42,58]. Moreover, COVID-19–related aspects were considered in single studies. The COVID-19 stress level had a small negative correlation with satisfaction [58], and fear of COVID-19 was associated with positive views toward the transition to teletherapy but was not significantly associated with overall satisfaction scores [48].

#### Facilitating Conditions

One study examined the relationship between facilitating conditions and patient satisfaction with telemental health services. Ceniti et al [42] reported that the frequency of internet use was not significantly associated with patient satisfaction.

#### Effort and Performance Expectancy

None of the included studies examined the relationship between effort or performance expectancy and patient satisfaction with telemental health services.

## Discussion

### Principal Findings

#### Overview

This systematic review aimed to provide an extensive overview of the literature on and highlight the influential determinants of patient use and satisfaction with synchronous telemental health services during the COVID-19 pandemic. Various determinants of patient use and satisfaction were considered. Sociodemographic characteristics were most frequently examined. Nevertheless, health- and service-related determinants also received considerable attention. Major dimensions of the UTAUT, such as effort and performance expectancy, were neglected in recent studies. Although most associations were mixed or nonsignificant, some indications for potential relationships were found (eg, for sex, age, and symptom severity). This systematic review is the first to examine the determinants of patient use and satisfaction with synchronous telemental health services during the pandemic, thus markedly extending our current knowledge.

#### Sociodemographic Factors

Regarding sociodemographic factors, a variety of determinants were observed in the included studies. Most studies found that sex was not significantly associated with patient use and satisfaction. However, some studies with large samples found that female participants were more likely to use telemental health services. This suggests that previous findings regarding greater use of mental health services among female participants may also apply to the field of telemental health [66-69]. Moreover, this could explain the finding that women were less likely to go from low to either moderate or high telemedicine use [41], as they already had higher use rates before the occurrence of the pandemic.

When looking at patient age, mostly nonsignificant associations with the outcomes were found. Nevertheless, some large-sample studies found that older age was negatively associated with the outcomes and that older patients were more likely to use audio-only services compared with video services. This could be not only because of the lower likelihood of older adults using mental health care services [70] but also because of the digital divide in mobile health [71]. However, audio-only formats seem to be a promising alternative to video consultations for older adults, which was also found in other telemedicine areas during the pandemic (eg, academic medical center outpatient visits and oncological care) [72-74].

Race, ethnicity, area lived in (ie, rurality and province lived in), education, and other determinants (eg, refugee status, financial status, and living situation) were observed in only few studies and led to mainly nonsignificant or mixed associations with the outcomes. More research regarding these sociodemographic determinants is needed in the future. In summary, sociodemographic factors tend to play a role in patient use of telemental health services. In particular, sex and age appear to be potential determinants that were frequently observed. For patient satisfaction, mainly nonsignificant or mixed findings were reported.

#### Health Factors

Regarding health factors, symptom severity was observed in some studies and was mostly associated with lower use rates in patients with mental health conditions. This is in contrast to in-person mental health services research, where symptom severity was associated with an increased likelihood of seeking treatment [69]. A potential reason for this could be that patients with very severe symptoms were preferably kept in an in-person setting despite the pandemic to assure appropriate treatment. However, findings on engagement or attendance were mixed, with some studies suggesting that more severe symptoms were associated with an increased frequency of telemental health visits. This could mean that individuals with more severe symptoms were less likely to start teletherapy, but once they were participating in telemental health services, they used it more frequently than patients with less severe symptoms. For satisfaction, most of the associations were nonsignificant. In conclusion, the associations with determinants were mostly observed for patient use. Although psychological symptom severity seemed to be negatively associated with the likelihood of telemental health service use, some indications for a positive association with use frequency were observed.

#### Service Factors

With regard to service factors, various determinants were observed in different studies. For patient use, there was great heterogeneity in the observed aspects. Therefore, it is challenging to compare the results of these studies. More research in this field is clearly needed. Nevertheless, services that were delivered in video format seemed to be associated with higher patient satisfaction than services delivered via telephone. A qualitative study in primary care highlighted potential reasons for the preference of video services, including nonverbal cues and reassurance, lower risk of miscommunication, more personal experience, and increased focus [75]. A recent systematic review on using telephone and video services for mental health treatment also emphasized the strengths of the video format [76]. However, they also stated that the telephone format can be superior to the video format in some cases (eg, fewer technological challenges [76]).

#### Experience

With regard to the experience with telemental health services, previous engagement in mental health services was not significantly associated with patient use. This could potentially mean that telemental health use rather depends on need factors than on experience. Regarding patient satisfaction, findings for the treatment duration were mixed. However, the number of telehealth sessions attended seemed to be associated with fewer missed sessions and higher satisfaction ratings. Therefore, patients might have got used to the new situation over time and had adapted to the remote format.

#### Psychosocial Influence

With regard to psychosocial factors, no determinants of patient use were observed. For patient satisfaction, significant determinants were only observed in single studies. Further research, including on psychosocial determinants, is urgently required. Especially factors such as personality (eg, neuroticism or conscientiousness) and social determinants (eg, loneliness) could be of interest for the future of telemental health, considering their impact on health care use [77,78].

#### Facilitating Conditions

With regard to facilitating conditions, the health insurance type was not significantly associated with patient use in some studies. The frequency of internet use was also not significantly associated with patient satisfaction in 1 single study. More research is needed in this area to identify potential facilitators of telemental health use and satisfaction.

#### Effort and Performance Expectancy

With regard to effort and performance expectancy, no study included determinants from these constructs. Considering that these 2 dimensions are key elements of the UTAUT, future research should urgently include determinants from this area.

### Study Quality

Overall, the quality of the included studies was mainly good or fair and did not vary substantially between the different studies. Most studies included large samples and some included even very large electronic medical record data sets [41,44]. However, the generalizability of our results is limited considering that the evidence mainly came from North America and Western countries and because of differences in psychiatric care and telemental health services. Most studies did not provide participation rates, sample size justification, power description, or variance and effect estimates, which are important information sources for the interpretation of the associations and the detection of potential biases (eg, selection bias).

### Future Research

Considering the findings of our systematic review, multiple research gaps were identified. In general, the inclusion of theoretical models is needed in future studies to set a more consistent focus on important determinants and to assure comparability of the studies. Future research should consider different types of use behavior (eg, frequency of use, adoption, and attendance) and satisfaction (different scales or areas). Established scales should be used to measure the outcomes rather than single items (especially for satisfaction) because single items are more prone to bias. Moreover, to improve the understanding of the relationships between the different determinants and their effects on patient use and satisfaction, future studies that examine the influencing chain and process behind the outcomes are needed. In addition, future studies should explore whether certain telemental health formats (eg, telephone, video, or asynchronous formats) are especially suited for the treatment of specific diagnoses (eg, depression, anxiety, or schizophrenia). Furthermore, longitudinal studies are needed to verify the findings and test for potential changes over time. Longitudinal studies are also of interest to see whether findings regarding use and satisfaction during the pandemic also apply to postpandemic circumstances. For instance, a recent qualitative study found that remote services were only seen as a good alternative to in-person mental health services during extreme circumstances [79]. Additional qualitative research is needed, for example, to explore the barriers of users who do not indicate high use or satisfaction rates to make telemental health services more accessible and user friendly in the future.

With regard to the UTAUT dimensions, major research gaps were revealed. In particular, for the dimensions effort and performance expectancy, psychosocial influence and facilitating conditions research is missing in the respective literature. However, these dimensions could be valuable starting points for interventions, as they could potentially be influenced or adapted over time to improve use rates and satisfaction with telemental health services.

### Strengths and Limitations

Our systematic review was registered in PROSPERO and conducted in accordance with PRISMA guidelines to ensure the quality and transparency of the manuscript. A double-screening approach was used to screen 3 databases, which generally was found to be advanced in comparison with single screening and lead to fewer missed studies in the screening process [80]. In addition, data extraction and study quality assessment were performed by 2 reviewers. Furthermore, this review is the first to evaluate the existing literature on the determinants of use and satisfaction with synchronous telemental health services during the COVID-19 pandemic.

However, this study has some limitations. Only peer-reviewed quantitative studies were included. Therefore, potentially meaningful studies were not considered (eg, from the gray literature). Nevertheless, this step promoted the quality of the included studies and the comparability of the findings. In addition, only German and English language articles were screened, whereby relevant articles in other languages could have been missed. Finally, no meta-analysis was performed because of the high heterogeneity in study designs, outcomes, and effect measures.

### Conclusions

The extensive implementation of synchronous telemental health services during the pandemic triggered new research in this field. This systematic review was the first to synthesize studies that observed the determinants of patient use and satisfaction with these services. Significant heterogeneity was observed among the included studies. The findings revealed potential target groups (eg, female and young patients with mild symptoms) for future postpandemic telemental health interventions. However, the findings also revealed that patient groups that were especially burdened during the pandemic (such as older patients with severe symptoms) were harder to reach, and efforts are required to address such groups. Finally, knowledge gaps in the recent literature were highlighted, which call for future quantitative and qualitative research to secure and expand the recent findings. This could help to better understand barriers as well as individual preferences and eventually improve telemental health services in the future.

## Appendix

Multimedia Appendix 1 List of all included studies in the narrative synthesis.

## Abbreviations

PRISMA: Preferred Reporting Items for Systematic Reviews and Meta-Analyses
UTAUT: Unified Theory of Acceptance and Use of Technology
