# Supplementary material for: Determinants of Patient Use and Satisfaction With Synchronous Telemental Health Services During the COVID-19 Pandemic: Systematic Review
Source: JMIR Ment Health. 2023 Aug 18;10:e46148. doi: 10.2196/46148 (PMC10474517; doi:10.2196/46148)
Supplement: Multimedia Appendix 1 [file mental_v10i1e46148_app1.docx]

**List of all included studies in the narrative synthesis**

41. Ainslie M, Brunette MF, Capozzoli M. Treatment Interruptions and Telemedicine Utilization in Serious Mental Illness: Retrospective Longitudinal Claims Analysis. *JMIR Mental Health*. 2022;9(3):e33092. doi:10.2196/33092

42. Ceniti AK, Abdelmoemin WR, Ho K, et al. "One Degree of Separation": A Mixed-Methods Evaluation of Canadian Mental Health Care User and Provider Experiences With Remote Care During COVID-19. *The Canadian Journal of Psychiatry*. 2022;67(9):712-722. doi:10.1177/07067437211070656

43. Chakawa A, Belzer LT, Perez-Crawford T, Yeh HW. COVID-19, Telehealth, and Pediatric Integrated Primary Care: Disparities in Service Use. *Journal of Pediatric Psychology*. 2021;46(9):1063-1075. doi:10.1093/jpepsy/jsab077

44. Connolly SL, Stolzmann KL, Heyworth L, et al. Patient and provider predictors of telemental health use prior to and during the COVID-19 pandemic within the Department of Veterans Affairs. *American Psychologist*. 2021;77(2):249-261. doi:10.1037/amp0000895

45. Guinart D, Marcy P, Hauser M, Dwyer M, Kane JM. Patient attitudes toward telepsychiatry during the COVID-19 pandemic: A nationwide, multisite survey. *JMIR Mental Health* 2020;7(12):e24761. doi:10.2196/24761

46. Haxhihamza K, Arsova S, Bajraktarov S, et al. Patient satisfaction with use of telemedicine in university clinic of psychiatry: Skopje, north Macedonia during COVID-19 pandemic. *Telemedicine and e-Health*. 2021;27(4):464-467. doi:10.1089/tmj.2020.0256

47. Hutchison M, Russell BS, Gans KM, Starkweather AR. Online administration of a pilot mindfulness-based intervention for adolescents: Feasibility, treatment perception and satisfaction. *Current Psychology*. 2022; doi:10.1007/s12144-022-03025-x

48. Lewis YD, Elran-Barak R, Tov RGS, Zubery E. The abrupt transition from face-to-face to online treatment for eating disorders: a pilot examination of patients' perspectives during the COVID-19 lockdown. *Journal of Eating Disorders*. 2021;9(1):31. doi:10.1186/s40337-021-00383-y

49. Lohmiller J, Schaeffeler N, Zipfel S, Stengel A. Higher Acceptance of Videotelephonic Counseling Formats in Psychosomatic Medicine in Times of the COVID-19 Pandemic. *Frontiers in Psychiatry*. 2021;12:747648. doi:10.3389/fpsyt.2021.747648

50. Lynch DA, Stefancic A, Cabassa LJ, Medalia A. Client, clinician, and administrator factors associated with the successful acceptance of a telehealth comprehensive recovery service: A mixed methods study. *Psychiatry Research*. 2021;300:113871. doi:10.1016/j.psychres.2021.113871

51. Meininger L, Adam J, von Wirth E, et al. Cognitive-behavioral teletherapy for children and adolescents with mental disorders and their families during the COVID-19 pandemic: a survey on acceptance and satisfaction. *Child and Adolescent Psychiatry and Mental Health*. 2022;16(1):61. doi:10.1186/s13034-022-00494-7

52. Michaels TI, Singal S, Marcy P, et al. Post-acute college student satisfaction with telepsychiatry during the COVID-19 pandemic. *Journal of Psychiatric Research*. 2022;151:1-7. doi:10.1016/j.jpsychires.2022.03.035

53. Miu AS, Vo HT, Palka JM, Glowacki CR, Robinson RJ. Teletherapy with serious mental illness populations during COVID-19: telehealth conversion and engagement. *Counselling Psychology Quarterly*. 2021;34(3-4):704-721. doi:10.1080/09515070.2020.1791800

54. Morgan AA, Landers AL, Simpson JE, et al. The transition to teletherapy in marriage and family therapy training settings during COVID-19: What do the data tell us? *J Marital Fam Ther*. 2021;47(2):320-341. doi:10.1111/jmft.12502

55. Nesset MB, Lauvrud C, Meisingset A, Nyhus E, Palmstierna T, Lara-Cabrera ML. Development of nurse-led videoconference-delivered cognitive behavioural therapy for domestic violence: Feasibility and acceptability. *Journal of Advanced Nursing*. 2022;00:1-10. doi:10.1111/jan.15347

56. Severe J, Tang R, Horbatch F, Onishchenko R, Naini V, Blazek MC. Factors Influencing Patients’ Initial Decisions Regarding Telepsychiatry Participation During the COVID-19 Pandemic: Telephone-Based Survey. *JMIR Form Res*. 2020;4(12):e25469. doi:10.2196/25469

57. Sizer MA, Bhatta D, Acharya B, Paudel KP. Determinants of Telehealth Service Use among Mental Health Patients: A Case of Rural Louisiana. *International Journal of Environmental Research and Public Health*. 2022;19(11):6930. doi:10.3390/ijerph19116930

58. ter Heide F, de la Rie S, de Haan A, et al. Wellbeing and clinical videoconferencing satisfaction among patients in psychotrauma treatment during the coronavirus pandemic: Cross-sectional study. *European Journal of Psychotraumatology* 2021;12(1):1906021. doi:10.1080/20008198.2021.1906021

59. Tobin ET, Hadwiger A, DiChiara A, Entz A, Miller-Matero LR. Demographic Predictors of Telehealth Use for Integrated Psychological Services in Primary Care During the COVID-19 Pandemic. *Journal of Racial and Ethnic Health Disparities*. 2022;doi:10.1007/s40615-022-01334-2

60. Vakil T, Svenne DC, Bolton JM, Jiang D, Svenne S, Hensel JM. Analysis of the uptake and associated factors for virtual crisis care during the pandemic at a 24-h mental health crisis centre in Manitoba, Canada. *BMC Psychiatry*. 2022;22(1):527. doi:10.1186/s12888-022-04166-w
